# Novel microsatellite instability test of sebaceous tumours to facilitate low-cost universal screening for Lynch syndrome

**DOI:** 10.1093/ced/llaf005

**Published:** 2025-01-23

**Authors:** Richard Gallon, Georgie Holt, Waleed Alfailakawi, Akhtar Husain, Claire Jones, Peter Sowter, Mauro Santibanez-Koref, Michael S Jackson, John Burn, Sam Cook, Neil Rajan

**Affiliations:** Translational and Clinical Research Institute, Faculty of Medical Sciences, Newcastle University, Newcastle upon Tyne, UK; Translational and Clinical Research Institute, Faculty of Medical Sciences, Newcastle University, Newcastle upon Tyne, UK; Translational and Clinical Research Institute, Faculty of Medical Sciences, Newcastle University, Newcastle upon Tyne, UK; Department of Pathology, Royal Victoria Infirmary, Newcastle upon Tyne, UK; Department of Pathology, Royal Victoria Infirmary, Newcastle upon Tyne, UK; Translational and Clinical Research Institute, Faculty of Medical Sciences, Newcastle University, Newcastle upon Tyne, UK; Biosciences Institute, Faculty of Medical Sciences, Newcastle University, Newcastle upon Tyne, UK; Biosciences Institute, Faculty of Medical Sciences, Newcastle University, Newcastle upon Tyne, UK; Translational and Clinical Research Institute, Faculty of Medical Sciences, Newcastle University, Newcastle upon Tyne, UK; Department of Pathology, Royal Victoria Infirmary, Newcastle upon Tyne, UK; Translational and Clinical Research Institute, Faculty of Medical Sciences, Newcastle University, Newcastle upon Tyne, UK; Department of Dermatology, Royal Victoria Infirmary, Newcastle upon Tyne, UK

## Abstract

**Background:**

One in five patients with sebaceous tumours (STs) may have Lynch syndrome (LS), an inherited disorder that increases the risk of developing cancer. Patients with LS benefit from cancer surveillance and prevention programmes and immunotherapy. While universal tumour mismatch repair (MMR) deficiency testing is recommended in colorectal and endometrial cancers to screen for LS, there is no consensus screening strategy for STs, leading to low testing rates and inequity of care.

**Objectives:**

To assess a low-cost and scalable sequencing-based microsatellite instability (MSI) assay, previously shown to enhance LS screening of colorectal cancers, for MMR deficiency detection in STs against the current clinical standard of immunohistochemistry (IHC).

**Methods:**

Consecutive ST cases (*n* = 107) were identified from the records of a single pathology department. MMR protein IHC staining was interpreted by a consultant histopathologist. MSI analysis used amplicon sequencing of 14 microsatellites and a naive Bayesian classifier to calculate the sample MSI score.

**Results:**

Loss of MMR protein expression was observed in 49/104 STs with interpretable IHC [47.1%, 95% confidence interval (CI) 37.3–57.2]. MMR deficiency was less frequent in carcinoma than in adenoma and sebaceoma (*P* = 4.74 × 10^–3^). The majority of MMR-deficient STs had concurrent loss of MSH2 and MSH6 expression. The MSI score achieved a receiver operator characteristic area under curve of 0.944 relative to IHC. Lower MSI scores were associated with MSH6 deficiency.

**Conclusions:**

These data support MSI testing as an adjunct or alternative to MMR IHC in STs. Integration of STs into established LS screening pathways using this high-throughput methodology could increase testing and reduce costs.

## Graphical Abstract

**Graphical Abstract:**
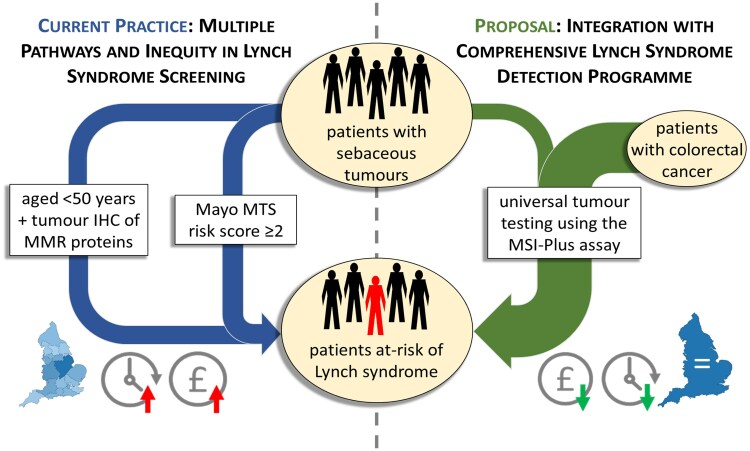

## Teaser

Lynch syndrome (LS) screening using reflex tumour mismatch repair (MMR) deficiency testing is recommended in all colorectal and endometrial cancers but not in sebaceous tumours (STs) despite LS being more common among patients with STs. A novel MMR deficiency test for STs, the Newcastle MSI-Plus assay, demonstrated equivalent diagnostic accuracy to the clinical standard immunohistochemistry (IHC) test. The MSI-Plus assay is in clinical service in northern England for colorectal cancers and is cheaper and more scalable than IHC. Integration of ST testing with these established screening pathways could streamline service provision, reduce testing inequity, make healthcare cost savings and, ultimately, increase LS diagnoses.

**What is already known about this topic?**

- Tumour mismatch repair (MMR) deficiency testing can be used to screen for Lynch syndrome (LS), an inherited disorder that increases the risk of developing cancer.
- Universal testing for LS is recommended for colorectal and endometrial cancers but not for sebaceous tumours (STs), despite LS being sixfold more prevalent among patients with STs.
- Only 34% of sebaceous carcinomas were tested for LS in England in 2018, highlighting a disparity in LS screening between visceral and cutaneous malignancies.

**What does this study add?**

- We assessed a novel, low-cost and scalable MMR deficiency test for sebaceous tumours, the Newcastle MSI-Plus assay.
- The MSI-Plus assay demonstrated equivalence as an MMR deficiency test compared with immunohistochemistry, the clinical standard.
- MSH6-deficient tumours had lower microsatellite instability, consistent with other tumour types.
- STs could be integrated with established LS screening pathways already using the MSI-Plus assay to streamline service provision, increase testing and reduce costs.

Sebaceous tumours (STs) are a rare group of skin tumours of the sebaceous gland, encompassing sebaceoma, adenoma and carcinoma.^1^ STs are strongly associated with the cancer predisposition syndrome, Lynch syndrome (LS), with up to one in five patients with STs having LS.^2^ Screening of STs for LS is not routinely performed in every case, and variation in practice has been highlighted, suggesting inequity of care.^3^ Notably, reflex mismatch repair (MMR) deficiency testing of all colorectal cancers (CRCs) and all endometrial cancers (ECs) to screen for LS is recommended as a more sensitive and more cost-effective approach than using clinical criteria.^4–7^ A recent meta-analysis found that > 95% of all types of STs from patients with LS (*n* = 177) were MMR deficient,^8^ suggesting MMR deficiency testing of STs may have a higher sensitivity for LS screening than the 80–85% previously reported on which current screening guidelines that use clinical criteria are based.^9–11^ These observations, together with the potential benefit from integration with established pipelines for CRC and EC, suggest reflex MMR deficiency testing of STs could improve LS detection, as has recently been called for by an expert and patient group in the UK.^12^

LS (OMIM #609310, #120435, #614350 and #614337) is diagnosed in individuals carrying a constitutional (germline) pathogenic variant affecting one of four genes (*MLH1*, *MSH2*, *MSH6* or *PMS2*) that constitute the core MMR system. It is associated with gastrointestinal and genitourinary tract cancers, as well as STs and other rare tumour types.^4,5^ MMR deficiency is characteristic of LS tumours and, hence, is a useful biomarker in LS screening. Once identified, patients with LS benefit from personalized healthcare and cancer risk management, including immune checkpoint inhibitor therapy, genetic counselling, surveillance, prophylactic surgery and daily acetylsalicylic acid (aspirin) intake.^4,5,13,14^

Immunohistochemistry (IHC) is the clinical standard used for MMR deficiency testing of STs. Microsatellite instability (MSI) analysis, the detection of insertion–deletion mutations in repetitive microsatellite DNA sequences, is an alternative assay for MMR deficiency but is rarely used in ST.^3^ IHC and newer next-generation sequencing (NGS)-based MSI assays have not been compared in unselected cases of ST at scale. MSI assays have the advantage of offering a functional readout of MMR status to avoid false-negative results due to missense (and other) MMR variants that disrupt function but not expression,^15,16^ while NGS offers scalable and automatable protocols and analysis to reduce the burden of testing on pathologists’ and scientists’ time. The Newcastle MSI-Plus assay, a novel amplicon-sequencing MSI and mutation hotspot assay, has recently been deployed in National Health Service (NHS) diagnostic laboratories in the north of England for CRC testing. In its first year, it tested > 95% of the approximately 2500 CRC diagnoses, halved testing turnaround times and doubled detection of patients at risk of LS compared with previous years.^17^ Because of its low cost and scalability, the assay has the capacity to cover additional tumour types such as ST. Therefore, we investigated the sensitivity and specificity of the Newcastle MSI-Plus assay in ST compared with IHC, with a view to harmonizing LS screening of patients with ST across established clinical pathways.

## Materials and methods

### Samples

Patients with STs diagnosed between November 2012 and March 2021 with available formalin-fixed paraffin-­embedded (FFPE) tissue were identified through the NovoPath Cellular Pathology Research Department of the Royal Victoria Infirmary, The Newcastle upon Tyne Hospitals NHS Foundation Trust (Table S1; see Supporting Information). STs were classified as sebaceoma, adenoma or carcinoma by a consultant histopathologist using haematoxylin and eosin-stained tissue sections and World Health Organization classification criteria.^18^

### Haematoxylin and eosin staining and mismatch repair protein immunohistochemistry

Haematoxylin and eosin staining and MMR protein IHC of FFPE tissue sections were performed by NovoPath using the Dako CoverStainer (Agilent Technologies, Santa Clara, CA, USA) and Ventana Discovery Ultra automated IHC staining platform (Ventana Medical Systems, Basel, Switzerland), respectively. Detailed protocols are provided in Appendix S1 (see Supporting Information). Stained sections were viewed using a Leica DM2000 microscope (Leica Microsystems, Milton Keynes, UK). MMR protein IHC was interpreted by a consultant histopathologist. Images were captured with a Nikon DS-Fi1-U2 (Nikon Instruments, Melville, NY, USA) at × 400 magnification.

### DNA extraction

Genomic DNA was extracted from 10-μm tissue curls using the Qiagen GeneRead DNA FFPE Kit (Qiagen, Hilden, Germany). DNA concentrations were quantified using a QuBit 3 Fluorometer (Invitrogen, Waltham, MA, USA).

### Newcastle MSI-Plus assay

The Newcastle MSI-Plus assay followed the standard protocols for clinical diagnostic testing of CRCs in the Northern Genetics Service, The Newcastle upon Tyne Hospitals NHS Foundation Trust.^17^ In brief, 14 tissue agnostic, highly sensitive MSI markers^19,20^ were amplified and tagged with Illumina sequencing adapters, sample indexes and custom sequencing primer binding sites (Illumina, San Diego, CA, USA) using multiplex polymerase chain reaction. Amplicons were sequenced to approximately × 2000 read depth on a MiSeq (Illumina). Reads were aligned to the human reference genome hg19 using BWA version 0.7.17 mem^21^ and microsatellite allele frequencies extracted using R version 4.2.2 (R Foundation for Statistical Computing, Vienna, Austria), as previously described.^19^ An MSI score was calculated for each sample using a Bayesian classification method, based on the frequency and allelic bias of microsatellite deletions,^19^ trained on data from a published CRC cohort.^17^ An MSI score > 0 indicates a sample is MSI-high (MSI-H; MMR deficient). An MSI score < 0 indicates a sample is microsatellite stable (MSS; MMR proficient). An MSI score passed quality control with a median MSI marker read depth ≥ 100, and scores between –5 and +5 required a repeat assay to confirm the classification, according to clinically established criteria.^17^

### Mismatch repair gene sequencing and variant detection

MMR genes were sequenced using molecular inversion probe (MIP) amplicon sequencing.^22^ In total, 214 MIPs capturing *MLH1*, *MSH2*, *MSH6* and *PMS2* exons (Table S2; see Supporting Information) were designed using MIPgen.^23^ MIPs (synthesized by Metabion, Planegg, Germany) were pooled, 5ʹ-phosphorylated using T4 Polynucleotide Kinase (New England Biolabs, Ipswich, MA, USA), and diluted to 0.1 nmol L^–1^ each as previously described.^24^ MIP amplification of sample DNAs, library generation and sequencing to approximately × 1500 read depth followed published protocols.^24^ Reads were aligned to the human reference genome hg19 using BWA version 0.7.17 mem.^21^ Binary alignment and map (BAM) files were generated using SAMtools version 1.9.^25^ BAMclipper removed the MIP targeting arm sequence from reads.^26^ Variants were called using two variant callers from GATK version 4.6.0.0,^27^ to maximize variant detection, HaplotypeCaller and Mutect2, with ‘–max-reads-per-alignment-start’ set to 0 and ‘–dont-use-soft-clipped-bases’ set to true. Variants were annotated using Ensembl Variant Effect Predictor version 102.0,^28^ including SIFT and PolyPhen prediction of missense variant pathogenicity. Variants were filtered using R version 4.2.2, the vcfR package^29^ and custom thresholds (Appendix S1), and checked in ClinVar.^30^

### Data analysis and statistics

Analyses used R version 4.2.2 and packages ‘ggplot2’^31^ and ‘pROC’.^32^ Frequencies and sensitivity and specificity estimates were given as 95% confidence intervals (CI) based on a Clopper–Pearson binomial distribution. A Fisher’s exact test was used to assess associations in counts across two categorical variables. A Mann–Whitney *U*-test or Kruskal–Wallis test was used to assess associations between a continuous variable and categorical variable of two or more groups, respectively. Receiver operator characteristic area under the curve (ROCauc) was calculated using the ‘pROC roc()’ function. All statistical tests were two-sided.

## Results

### Description of cohort of patients with sebaceous tumours

In total, 110 consecutive STs from distinct patients (Table S1; see Supporting Information), diagnosed between November 2012 and March 2021, were available for MSI testing and MMR protein IHC. Histological review identified 33 sebaceoma, 61 adenoma and 13 carcinoma. Three samples were found to have an ambiguous, likely nonsebaceous, tissue of origin or to not contain any tumour cells, and were excluded. Of the 107 patients with histologically confirmed ST, 64 were men and 43 were women. The median age at diagnosis was 77 years (range 29–97). There was no association between tumour type and patient age (*P* = 0.10) or sex (*P* = 0.26).

### Almost half of sebaceous tumours show loss of mismatch repair protein expression

Each ST was analysed by IHC of all four MMR proteins to provide a reference result against which the sensitivity and specificity of MSI analysis could be determined. Three samples, one sebaceoma and two adenomas, failed to give interpretable IHC staining because of issues with sample processing. Of the 104 STs with interpretable IHC, 52 (50.0%, 95% CI 40.0–60.0) retained MMR protein expression, observed as uniform nuclear expression with slight variation dependent on cell proliferative activity. Forty-nine STs (47.1%, 95% CI 37.3–57.2) showed a clear loss of expression of at least one MMR protein, observed as complete negative staining in tumour cells in most cases, but also very weak or very focal staining. The three remaining STs (2.9%, 95% CI 0.6–8.2), all of which were carcinoma, had equivocal staining in one or more MMR proteins leading to uncertain MMR status. There was no association between IHC classification and patient age (*P* = 0.26) or sex (*P* > 0.99). Unequivocal MMR expression loss was associated with tumour type (*P* = 4.74 × 10^–3^), with more frequent loss among sebaceoma (*n* = 14/32; 44%, 95% CI 26–62) and adenoma (*n* = 32/59; 54%, 95% CI 41–67) than carcinoma (*n* = 3/13; 23%, 95% CI 5–54] (Table 1).

**Table 1:** Mismatch repair protein immunohistochemistry (IHC) classification compared with sebaceous tumour type

IHC classification	Tumour type (*n* = 107)		
	Sebaceoma	Adenoma	Carcinoma
MMRd	14	32	3
Equivocal	0	0	3
MMRp	18	27	7
NA	1	2	0

MMRd, mismatch repair deficient; MMRp, mismatch repair proficient; NA, not applicable due to uninterpretable technical artefacts.

The MMR proteins form two heterodimers, MutSα (composed of MSH2 and MSH6) and MutLα (composed of MLH1 and PMS2). MSH2 and MLH1 are essential for heterodimer stabilization, so loss of their expression leads to concurrent loss of MSH6 or PMS2 staining, respectively.^33,34^ Concurrent loss of MSH2 and MSH6 expression was observed in the majority [*n* = 27/49 (55%), 95% CI 40–69] of MMR-deficient STs. Isolated loss of MSH6 and of PMS2 expression can be seen as MSH2 and MLH1 can be stabilized by alternate heterodimers.^33,34^ The isolated loss of MSH6 was observed in only 4 of the 49 cases (8%, 95% CI 2–20), one of which also had equivocal staining for MSH2.

**Figure 1:**
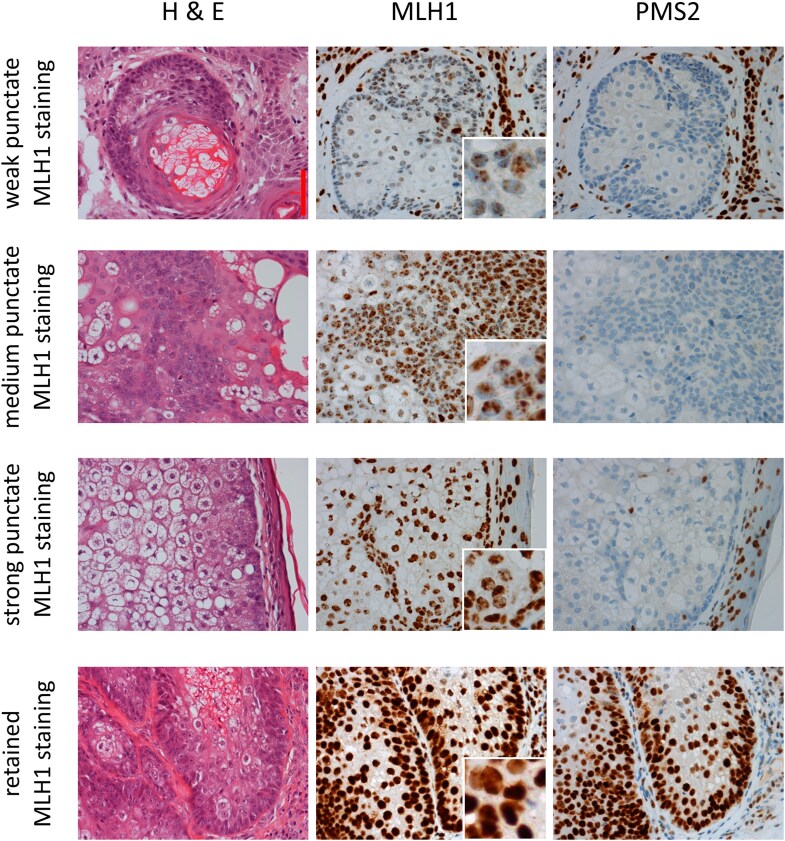
Examples of variable immunohistochemistry (IHC) staining patterns of MLH1 from four sebaceous tumours ranging from retained MLH1 staining to weak punctate staining (middle panels). Haematoxylin and eosin staining (H&E; left panels) and PMS2 IHC (right panels) are also shown for each tumour. Images were captured with a Nikon DS-Fi1-U2 at × 400 magnification. The red scale bar (top left panel) represents 50 μm.

The interpretation of MLH1 expression was complicated by punctate staining patterns of varying intensity. Across 16 cases of potential MLH1 loss, 10 were considered to have equivocal MLH1 staining because of a strong punctate pattern, while the other 6 were considered to have loss of MLH1 expression with weak-to-medium punctate staining (Figure 1). Interpretation was supported by analysis of PMS2 expression: 15 of the 16 STs with equivocal or absent MLH1 staining showed a clear absence of or weak PMS2 staining, suggesting loss of MLH1 expression. The other ST, a carcinoma with strong punctate (equivocal) MLH1 staining, showed equivocal PMS2 staining. Assuming strong punctate MLH1 staining with loss of PMS2 staining indicates loss of MLH1 expression,^35–38^ concurrent loss of MLH1 and PMS2 expression was observed in 15/49 MMR-deficient ST (31%, 95% CI 18–45), while isolated PMS2 expression loss was observed in only 1/49 (2%, 95% CI 0.1–11%). Less common patterns of MMR expression loss were found in the remaining two MMR-deficient STs (4%; 95% CI 0.5–14%), including absent staining of three or all four MMR proteins. There was no association between the pattern of expression loss and tumour subtype (*P* = 0.50), patient age (*P* = 0.94) or sex (*P* = 0.56).

### Microsatellite instability analysis is a sensitive and specific biomarker of mismatch repair deficiency in sebaceous tumours

All 107 STs were analysed by the Newcastle MSI-Plus assay. Forty-two STs were MSI-H (39.3%, 95% CI 30.0–49.2) and 64 were MSS (59.8%, 95% CI 49.9–69.2). One sample had an inconclusive classification (MSI score set to 0 after conflicting scores between –5 and +5) (0.9%, 95% CI 0.0–5.1). To estimate sensitivity and specificity of MSI analysis, MSI classification was compared with the IHC reference results for the 100 STs that had conclusive MSI and IHC classifications. MSI analysis achieved 83% sensitivity (95% CI 70–93) by classification of 40/48 STs with loss of MMR expression as MSI-H, with 100% specificity (95% CI 93–100%) by classification of 52/52 STs with retained MMR expression as MSS (Table 2). Of the eight STs with an MSS result discordant with the IHC reference, half had equivocal MLH1 staining with concurrent PMS2 loss, making this staining pattern over-represented among discordant cases (odds ratio 16.3; *P* = 1.89 × 10^–3^).

**Table 2:** Mismatch repair protein immunohistochemistry (IHC) staining pattern compared with microsatellite instability (MSI) classification by the Newcastle MSI-Plus assay in sebaceous tumours

IHC classification and staining pattern	MSI classification (*n* = 107)		
	MSS	Uncertain	MSI–H
MMRd			
MSH2/MSH6/MLH1/PMS2 loss	0	0	1
MSH2/MSH6/PMS2 loss	0	0	1
MSH2/MSH6 loss	1	0	26
MSH2 eq., MSH6 loss	0	1	0
MSH6 loss	2	0	1
MLH1/PMS2 loss	1	0	5
MLH1 eq., PMS2 loss	4	0	5
PMS2 loss	0	0	1
Equivocal			
MLH1/PMS2 eq.	1	0	0
PMS2 eq.	2	0	0
MMRp			
Retained	52	0	0
NA			
Uninterpretable	1	0	2

eq., equivocal; MMRd, mismatch repair deficient; MMRp, mismatch repair proficient; MSI-H, microsatellite instability high; MSS, microsatellite stable; NA, not applicable due to uninterpretable technical artefacts.

It was not possible to comprehensively resolve discordance between the MMR deficiency tests, but reasons for discordance, as well as the origins of equivocal staining, were explored using MMR gene sequencing. Four of six MMR-deficient control STs had one or more pathogenic variants (PVs) detected, with each being concordant with the pattern of MMR IHC staining. Four of the eight STs with discordant results had a PV detected matching the pattern of MMR expression loss, suggesting that at least some of these were MMR deficient and were misclassified by MSI analysis. These included two MSH6-deficient STs and one ST with concurrent loss of expression of MSH2 and MSH6. Only one of the four STs with equivocal (strong punctate) MLH1 staining, loss of PMS2 and discordant MSS classification had an MMR PV detected, an *MLH1* splice site variant c.790 + 1G > A (Appendix S2; see Supporting Information).

**Figure 2:**
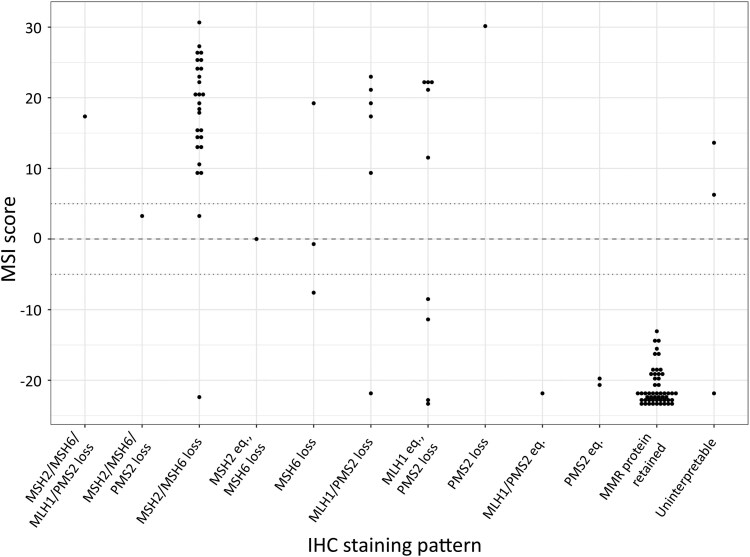
Mismatch repair (MMR) protein immunohistochemistry (IHC) staining pattern compared with microsatellite instability (MSI) scores generated by the Newcastle MSI-Plus assay in sebaceous tumours (*n* = 107). eq., equivocal.

ST MSI scores were further compared with the patterns of MMR protein expression (Figure 2). Three of the four MSH6-deficient STs were classified as MSS or had an inconclusive MSI score, and STs with concurrent loss of MSH2 and MSH6 had more positive MSI scores [median (range) +19.0 (–22.4 to +30.7)] than STs with isolated MSH6 loss [median –0.4 (–7.6 to +19.3); *P* = 0. 03]. A similar comparison was not made for the MutLα proteins given the complexities of MLH1 staining interpretation and the single case with isolated PMS2 loss. There was a trend for MSI scores closer to 0 among the MSS STs that had lost MMR expression [median –11.4 (–23.1 to –0.7)] compared with MSS STs that had retained MMR expression [median –21.8 (–23.5 to –13.1); *P* = 0.06], suggesting MSI classification could be improved using a different score threshold. Indeed, the MSI classifier was trained on data from CRCs and MSI is known to have a weaker signal in nongastrointestinal tumours.^39^ The ROCauc provides a less biased estimate of diagnostic accuracy. Here, MSI scoring achieved a very high ROCauc of 0.944 for the detection of unequivocal loss vs. retention of MMR protein expression.

## Discussion

Universal MMR deficiency testing of STs to identify patients for constitutional (germline) genetic testing has recently been called for by UK experts in partnership with the Lynch Syndrome UK patient group to harmonize screening practice with CRC and EC.^12^ Here, we assessed the viability of a novel testing strategy using MSI analysis and a low-cost, scalable assay – the Newcastle MSI-Plus assay – that is already in clinical practice for CRC testing in parts of England.^17^ Using IHC as the reference method, MSI scoring had a ROCauc of 0.944 and achieved 83% sensitivity and 100% specificity using a score threshold defined from CRCs. This suggests that MSI analysis is a viable adjunct or alternative MMR deficiency test to IHC for LS screening in STs, and warrants further study in clinical practice.

In this study, 49 of 104 STs (47.1%, 95% CI 37.3–57.2) were MMR deficient according to IHC, which falls in the middle of the broad range of 30–66% reported in the literature from similar large-scale studies of consecutive patients with ST.^40,41^ This broad range may reflect distinct IHC methodology and populations. The largest of these cohorts found an association between MMR deficiency and ST type, with increased frequency among adenoma and decreased frequency among sebaceoma and carcinoma.^41^ In contrast, we found adenoma and sebaceoma had comparable frequencies of MMR deficiency at 54% and 44%, respectively, but, in agreement, found carcinoma had the lowest frequency at 23%. Among MMR-deficient STs, the frequencies of IHC staining patterns are very similar between studies, with the majority having concurrent loss of MSH2 and MSH6 expression.^40,41^

MMR IHC staining of STs returned an ambiguous result in approximately 10% of the cases we studied. Punctate nuclear staining of MLH1 is a known staining pattern associated with the MLH1 M1 antibody used in this study,^35–38^ affecting as many as around 75% of tumours in other studies with the (potential) loss of MLH1 expression.^42^ In our study, punctate MLH1 staining was seen in each of the 16 STs with the (potential) MLH1 expression loss. Strong punctate staining presents a challenge to interpretation with a risk of erroneous classification as retained expression. A pragmatic approach is to regard any punctate MLH1 staining as defective/equivocal where there is supportive loss of PMS2 staining. However, this potentially reduces testing specificity, supported by the reduced concordance with MSI testing observed in these cases. The available material limited our ability to identify possible reasons for discordance (Appendix S2) and the MMR gene sequencing method used has limitations, in particular being insensitive to epigenetic variants, such as *MLH1* promoter methylation, that often disrupt MMR function.^43,44^ Therefore, the association between strong punctate MLH1 staining with PMS2 loss and discordant MSS classification remains unexplained. However, sequencing identified *MLH1* c.790+1G>A in one such ST and, interestingly, *MLH1* c.790+1G>A is a splice site variant that causes in-frame skipping of *MLH1* exons 9 and 10 and, therefore, a shortened protein that may retain antigenicity.^45^

Reduced MSI has been observed in MSH6-deficient tumours, even when exclusively analysing mononucleotide repeats (given MSH6 has no role in the repair of longer motif microsatellites such as di- or trinucleotide repeats), including CRC^46^ and EC.^47^ This is also seen in MSH6-deficient blood from patients with constitutional MMR deficiency,^20,48^ and is likely due to redundancy in repair between MutSα (MSH2–MSH6) and MutSβ (MSH2–MSH3) heterodimers.^33,34^ Three of four STs with isolated MSH6 deficiency had discordant MSS or uncertain classification and STs with isolated MSH6 loss had significantly lower MSI scores than STs with concurrent MSH2 and MSH6 loss, therefore showing the association of reduced MSI and MSH6 deficiency holds for ST.

In conclusion, MSI analysis is an effective MMR-deficiency test in ST and may reduce false-positive IHC results. In addition, MSI analysis can detect MMR deficiency where MMR protein expression is retained,^15,16^ although no such cases were identified in this study. Therefore, MSI assays may be used as adjunct or alternative MMR deficiency tests to IHC in ST, which would facilitate low-cost and integrated LS screening programmes in this currently overlooked tumour type.

## Supplementary Material

llaf005_Supplementary_Data

## Data Availability

FASTQ files generated by the Newcastle MSI-Plus assay and MMR gene MIP amplicon sequencing are available from the European Nucleotide Archive (https://www.ebi.ac.uk/ena/browser/home) using study IDs PRJEB79222 and PRJEB79223, respectively.
