# Supplementary material for: Novel microsatellite instability test of sebaceous tumours to facilitate low-cost universal screening for Lynch syndrome
Source: Clin Exp Dermatol. 2025 Jan 23;50(6):1155–62. doi: 10.1093/ced/llaf005 (PMC12099064; doi:10.1093/ced/llaf005)
Supplement: llaf005_Supplementary_Data [file llaf005_supplementary_data.zip › Supplementary Appendices.pdf]

Appendix S1 – Supplementary Methods

Haematoxylin and eosin staining – extended protocol

Haematoxylin and eosin (H&E) staining was performed by Novopath, The Newcastle upon Tyne Hospitals NHS Foundation Trust. Formalin-fixed paraffin-embedded (FFPE) tissue sections were cut at 4µm, mounted on Epredia™ SuperFrost Plus™ Adhesion slides (Thermo Fisher Scientific), and baked at 65°C for 10-15min. Staining was performed automatically by a Dako CoverStainer (Agilent Technologies). Sections were deparaffinised and rehydrated by the following steps: twice xylene (Genta Medical) for 3-4min, twice 99% industrial denatured alcohol (IDA) (Genta Medical) for 1-2min, once 70% IDA for 2min, and once tap water for 1min. Nuclei were stained by Dako Harris Haematoxylin (Agilent Technologies) for up to 2min, washed with distilled water for 1min, exposed to Dako Differentiation Solution (Agilent Technologies) for 1min, washed with tap water for 1min, exposed to Dako Bluing Buffer (Agilent Technologies) for 1min, washed with tap water for 1min, and dehydrated with 95% IDA for 1min. Additional tissue structures were stained using Dako Eosin Y Phloxine B (Agilent Technologies) for 2min, followed by acetified alcohol for 1min, and thrice dehydrated with 99% IDA for 1min. GentaClear (Genta Medical) solution was applied for 1min before glass cover-slipping using Dako Mounting Media (Agilent Technologies). H&E stained sections were viewed using a Leica DM2000 microscope (Leica).

Immunohistochemistry – extended protocol

MMR protein immunohistochemistry (IHC) was performed by Novopath, The Newcastle upon Tyne Hospitals NHS Foundation Trust. FFPE tissue sections from each sample were cut at 4µm, mounted on Epredia™ SuperFrost Plus™ Adhesion slides along with a control tissue section for MMR positive staining, and baked at 60°C for 60min. All staining was performed on the Ventana Discovery Ultra automated IHC staining platform based on recommended protocols and reagents and the Ventana VSS v12.5.4 software (Ventana Medical Systems). Sections were incubated for 8min with peroxidase-inhibitor (Discovery Inhibitor CM, Ventana Medical Systems) prior to staining. IHC primary and secondary antibodies are listed in Appendix S1 Table 1. Antibody dilutions used Da Vinci Green Diluent buffer (BioCare Medical). Primary antibody incubation times were: 24min, 12min, 36min, and 40min for MLH1, MSH2, MSH6, and PMS2, respectively. Following secondary antibody application, chromogen staining used the Optiview DAB and Optiview Amplification kits (Ventana Medical Systems) with 16min and 8min incubations, respectively. Hematoxylin counterstaining used recommended protocols and reagents (Ventana Medical Systems). IHC slides were mounted using a Leica CV5030 Automated Cover Slipper with Leica Mounting Media (Leica). Sections were viewed using a Leica DM2000 microscope and MMR protein staining was interpreted by a consultant histopathologist. Representative images of IHC staining were captured with a Nikon DS-Fi1-U2 at 400x magnification.

Mismatch repair gene sequencing – variant filtering

Filtering depended on the variant caller used: For HaplotypeCaller variant calls required QD ≥2 and MQ ≥40, and for Mutect2 required MMQ ≥40, and read depth ≥250. Variants were further filtered to have a VEP Impact of "HIGH" or to be a missense variant predicted to be "deleterious(_low_confidence)" by SIFT or "(possibly/probably)_damaging" by PolyPhen. Across sebaceous tumour samples analysed, many pathogenic variants with allele frequency <10% were detected that did not match the pattern of IHC staining. Given the age of the samples and formalin fixation, these were likely errors and, therefore, a minimum 10% variant allele frequency was used to filter variants

Antibody (type)	Manufacturer	Dilution	Source (clone)
Anti-MLH1 (primary)	Ventana Medical Systems	NA	Monoclonal mouse (M1)
Anti-MSH2 (primary)	Ventana Medical Systems	NA	Monoclonal mouse (G219-1129)
Anti-MSH6 (primary)	Abcam	1:200	Monoclonal rabbit (EPR3945)
Anti-PMS2 (primary)	Agilent Technologies	1:100	Monoclonal rabbit (EP51)
Anti-mouse (secondary, HRP-conjugated)	Ventana Medical Systems	NA	Discovery OmniMap anti-mouse HRP
Anti-rabbit (secondary, HRP-conjugated)	Ventana Medical Systems	NA	Discovery OmniMap anti-rabbit HRP

Appendix S1 Table 1: Immunohistochemistry antibodies.

Details of the primary and secondary antibodies used during mismatch repair protein immunohistochemistry. HRP – horse radish peroxidase.

Appendix S2 – MMR Gene Sequencing Results

Mismatch repair variant detection supports microsatellite instability analysis misclassifications

With limited or no residual sebaceous tumour sample available following microsatellite instability (MSI) analysis and mismatch repair (MMR) protein immunohistochemistry (IHC), it was not possible to comprehensively resolve discordance between the MMR deficiency test results, but it was of interest to assess possible reasons for discordance as well as the origins of equivocal staining. The eight discordant sebaceous tumours with MMR protein expression loss but microsatellite stable (MSS) classification, the three carcinoma with equivocal MMR staining and MSS classification, and an additional four MMR proficient controls (concordant retention of MMR protein and MSS classification) and six MMR deficient controls (concordant loss of MMR protein and MSI-high classification) were analysed for MMR gene variants using molecular inversion probe (MIP) amplicon sequencing (Hiatt et al. 2013; PMID: 23382536). For the detection of somatic variants down to 5% variant allele frequency (VAF), a minimum read depth of approximately 250x has been proposed (Jennings et al. 2017; PMID: 28341590). Here, a target read depth of 1500x was used to achieve this accounting for variation in MIP amplicon read depths (Hiatt et al. 2013; PMID: 23382536). Nearly all sebaceous tumours achieved a median read depth close to the target 1500x for *MLH1*, *MSH2*, and *MSH6*. *PMS2* read depths were lower, possibly due to older probe stocks or off-target amplification of *PMS2* pseudogenes (Appendix S2 Figure 1).

Four of the six MMR deficient controls had one or more pathogenic variants (PV) detected and, in each case, this was concordant with the pattern of MMR IHC staining (Appendix S2 Table 1). One of the four MMR proficient controls had a *PMS2* nonsense PV detected at 18.8% VAF (Appendix S2 Table 1), which could be incidental or an artefact given this sample had a particularly high burden of additional variants.

One of the three carcinoma with equivocal MMR IHC staining had a *PMS2* nonsense PV at 34.1% VAF, which may match with its equivocal PMS2 staining (Appendix S2 Table 1). Whilst nonsense PV would be expected to completely disrupt protein expression, it is possible a second, undetected *PMS2* variant accounts for the equivocal staining.

Four of the eight sebaceous tumours with discordant MMR deficiency test results had a PV detected with ≥10% VAF that matched with the pattern of MMR expression loss. Three had loss of MSH2 and MSH6 or MSH6 in isolation, and one had equivocal (strong punctate) MLH1 staining with loss of PMS2 staining (Appendix S2 Table 1). This supports that at least some of the discordant SN are MMR deficient and were misclassified by MSI analysis.

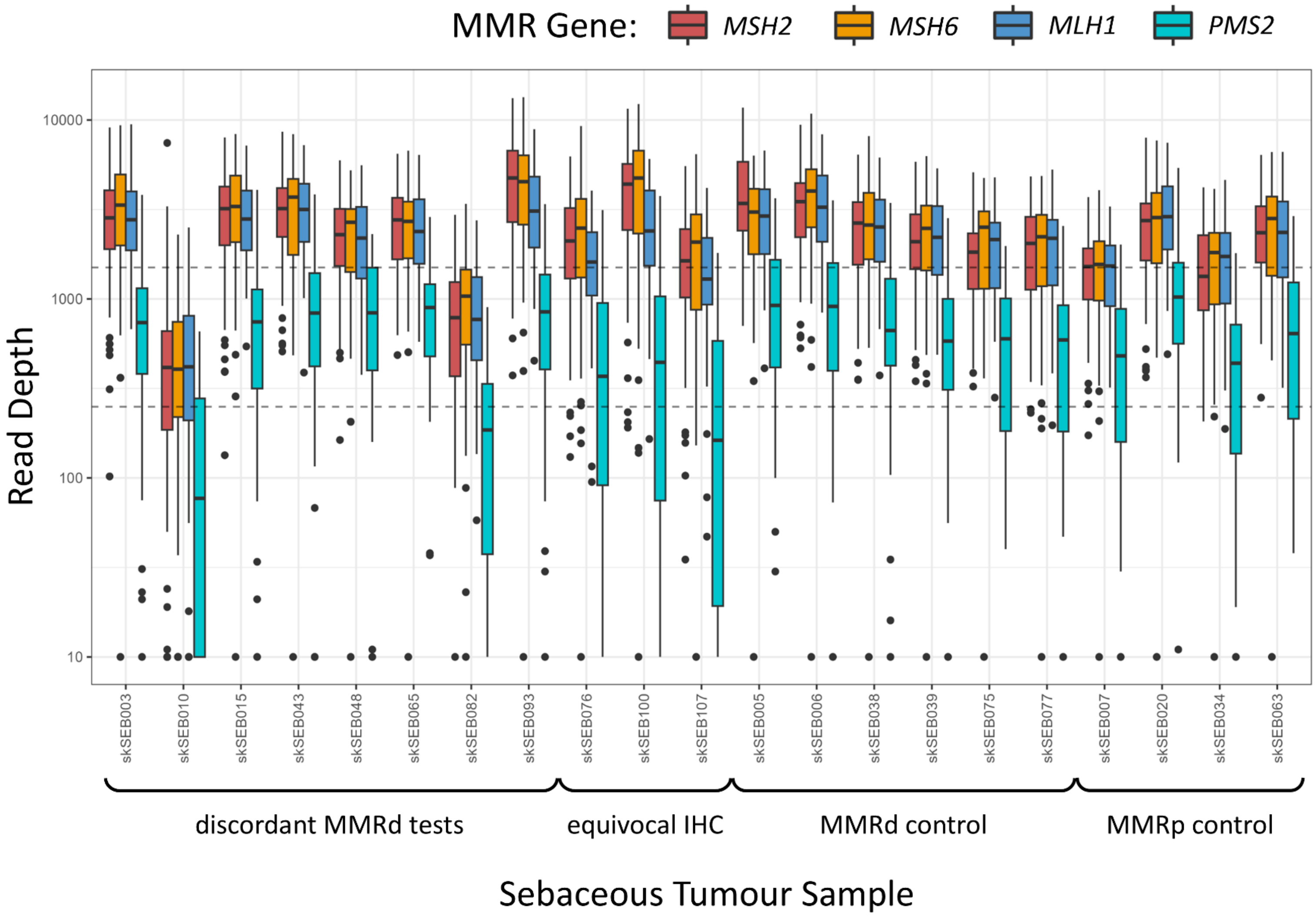

Appendix S2 Figure 1: Molecular inversion probe read depths. Molecular inversion probe read depths are shown for each mismatch repair (MMR) gene for each sebaceous tumour. The dashed lines are the minimum 250 and median 1500 read depth targets. MMRd – MMR deficient; MMRp – MMR proficient.

Group	Sample	Tumour Type	IHC Staining Pattern	MSI Score	Variant Summary	ClinVar Variant Reference	Variant Location	Variant cDNA	Variant Protein	Position Depth	Variant AF
Discordant	skSEB003	Sebaceoma	MSH6 loss	-7.6	*MSH6* frameshift PV	Variation ID: 89364: Accession: VCV000089364.95	2:48030647-48030648	*MSH6* c.3261_3262insC	MSH6 p.Phe1088Leufs	8030	0.432
					MSH6 nonsense PV	Variation ID: 2059416: Accession: VCV002059416.3	2:48033448	*MSH6* c.3752C>A	MSH6 p.Ser1251Ter	11612	0.147
	skSEB010	Sebaceoma	MLH1 eq., PMS2 loss	-8.5	*No high confidence PV calls*	*NA*	*NA*	*NA*	*NA*	*NA*	*NA*
	skSEB015	Sebaceoma	MLH1 eq., PMS2 loss	-11.4	*No high confidence PV calls*	*NA*	*NA*	*NA*	*NA*	*NA*	*NA*
	skSEB043	Adenoma	MLH1 eq., PMS2 loss	-21.8	*MLH1* splice donor PV	Variation ID: 90356; Accession: VCV000090356.39	3:37056036	*MLH1* c.790+1G>A	MLH1 p.?	6694	0.470
	skSEB048	Adenoma	MSH6 loss	-0.7	*MSH6* frameshift PV	Not reported	2:48028156-48028160	*MSH6* c.3034_3038del	MSH6 p.Glu1012fs	1534	0.522
					MSH6 frameshift PV	Not reported	2:48027105-48027106	*MSH6* c.1983_1984del	MSH6 p.Gly661fs	5319	0.102
	skSEB065	Carcinoma	MLH1 eq., PMS2 loss	-23.1	*No high confidence PV calls*	*NA*	*NA*	*NA*	*NA*	*NA*	*NA*
	skSEB082	Adenoma	MSH2/MSH6 loss	-22.4	*MSH2* frameshift PV	Not reported	2:47690290	*MSH2* c.1507delC	MSH2 p.Leu503fs	2224	0.112
	skSEB093	Carcinoma	MLH1 eq., PMS2 loss	-23.0	*No high confidence PV calls*	*NA*	*NA*	*NA*	*NA*	*NA*	*NA*
Equivocal	skSEB076	Carcinoma	PMS2 eq.	-19.8	*PMS2* nonsense PV	Not reported	7:6043397	*PMS2* c.277C>T	PMS2 p.Gln93Ter	504	0.341
	skSEB100	Carcinoma	MLH1/PMS2 eq.	-21.8	*No high confidence PV calls*	*NA*	*NA*	*NA*	*NA*	*NA*	*NA*
	skSEB107	Carcinoma	PMS2 eq.	-20.7	*No high confidence PV calls*	*NA*	*NA*	*NA*	*NA*	*NA*	*NA*
MMRd Control	skSEB005	Sebaceoma	MSH2/MSH6 loss	14.6	*No high confidence PV calls*	*NA*	*NA*	*NA*	*NA*	*NA*	*NA*
	skSEB006	Adenoma	MSH2/MSH6 loss	20.7	*MSH2* frameshift PV	Not reported	2:47702296-47702297	*MSH2* c.1892_1893insA	MSH2 p.Arg631fs	4646	0.611
					MSH6 frameshift PV	Variation ID: 89364; Accession: VCV000089364.95	2:48030639-48030640	*MSH6* c.3261_3262insC	MSH6 p.Phe1088Leufs	8134	0.238
	skSEB038	Carcinoma	MSH2/MSH6 loss	26.2	*MSH2* frameshift PV	Not reported	2:47702356	*MSH2* c.1952delT	MSH2 p.Ile651fs	4527	0.809
	skSEB039	Adenoma	MSH2/MSH6/PMS2 loss	3.0	*MSH2* frameshift PV	Not reported	2:47637410-47637411	*MSH2* c.544_545insT	MSH2 p.Asp182Valfs	6067	0.108
					PMS2 frameshift PV	Variation ID: 570785; Accession: VCV000570785.6	7:6027156-6027157	*PMS2* c.1239_1240insT	PMS2 p.Asp414Ter	421	0.394
	skSEB075	Adenoma	MLH1 eq., PMS2 loss	22.3	*MSH6* frameshift PV	Variation ID: 89363; Accession: VCV000089363.80	2:48030647	*MSH6* c.3261delC	MSH6 p.Pro1087fs	3803	0.234
					MLH1 delins 2nt splice donor (L)PV	Not reported*	3:37048554-37048555	*MLH1* c.453_453+1delinsAA	MLH1 p.?	4230	0.657
	skSEB077	Adenoma	MLH1 eq., PMS2 loss	21.0	*No high confidence PV calls*	*NA*	*NA*	*NA*	*NA*	*NA*	*NA*
MMRp Control	skSEB007	Adenoma	MMR protein retained	-16.5	*No high confidence PV calls*	*NA*	*NA*	*NA*	*NA*	*NA*	*NA*
	skSEB020	Carcinoma	MMR protein retained	-22.4	*No high confidence PV calls*	*NA*	*NA*	*NA*	*NA*	*NA*	*NA*
	skSEB034	Adenoma	MMR protein retained	-21.8	*PMS2* nonsense PV	Not reported	7:6043669	*PMS2* c.184G>T	PMS2 p.Gly62Ter	692	0.188
	skSEB063	Adenoma	MMR protein retained	-21.8	*No high confidence PV calls*	*NA*	*NA*	*NA*	*NA*	*NA*	*NA*

Appendix S2 Table 1: Mismatch repair gene pathogenic variants. Mismatch repair gene pathogenic variants detected in sebaceous tumours using molecular inversion probe amplicon sequencing. AF – allele frequency; eq. – equivocal staining (including strong punctate staining of MLH1); IHC – immunohistochemistry; (L)PV – likely pathogenic variant; MSI – microsatellite instability; PV – pathogenic variant. "Variant Location" chromosomal coordinates use human reference genome build hg19.
